# Using Taguchi Method to Determine the Optimum Conditions for Synthesizing Parapyruvate

**DOI:** 10.3390/molecules27061870

**Published:** 2022-03-14

**Authors:** Inn Lee, Nae-Cherng Yang

**Affiliations:** 1 Department of Nutrition, Chung Shan Medical University, Taichung 402, Taiwan; agnes09080913@gmail.com; 2 Department of Nutrition, Chung Shan Medical University Hospital, Taichung 402, Taiwan

**Keywords:** parapyruvate, KGDHC inhibitor, Taguchi’s L9 orthogonal array, optimal conditions, yield

## Abstract

The synthesis of parapyruvate is important for the analysis of the content in the pyruvate supplements and the study of aging-related neurodegenerative diseases. However, the pure parapyruvate crystal is not, as yet, commercially available. In this study, we applied the Taguchi’s L9 orthogonal array to investigate the optimal conditions for the preparation of the pure parapyruvate by the alkaline treatment of the pyruvic acid and then followed it with the solvent crystallization steps. We were also interested in revealing the major factors that affect the yield for the synthesized pure parapyruvate crystals. In addition, the parapyruvate-inhibited enzyme kinetic of α-ketoglutarate dehydrogenase complex (KGDHC) was also investigated. We found that the pure parapyruvate could be obtained in combination with an alkaline treatment and two solvent crystallization steps. The main factors affecting the yield of the pure parapyruvate were the concentration of the pyruvic acid (the reactant), the pH of the alkali treatment, the type of solvent used for the crystallization and the volume ratio of solvent used for crystallization. Finally, the optimal conditions could prepare parapyruvate crystals with a high purity of 99.8% and a high yield of 72.8%. In addition, the results demonstrate that parapyruvate is a reversibly competitive inhibitor for KGDHC.

## 1. Introduction

Parapyruvate is a dimer formed by the aldol addition of two molecules of pyruvate [1]. It is a common impurity in the commercial dietary supplements of pyruvate [2]. Parapyruvate is an inhibitor of the α-ketoglutarate dehydrogenase complex (KGDHC) and can be used as an inhibitor of the tricarboxylic acid cycle (TCA) cycle [3]. In our previous study, we published that parapyruvate can inhibit the activity of KGDHC so as to induce the senescence of the Hs68 fibroblasts [2]. Several studies suggest that a decreased KGDHC activity is involved in several aging-related neurodegenerative disorders such as Alzheimer’s disease [4,5,6,7,8,9] and Parkinson’s disease [10,11,12,13]. Thus, the synthesis of parapyruvate is important for an analysis of the content in the pyruvate supplements and for studying aging-related neurodegenerative diseases. However, the pure parapyruvate is not, as yet, commercially available. Previously, we referred to the alkaline-treatment method proposed by Margolis and Coxon [14] and developed a method to obtain the high-purity crystals of mono-potassium parapyruvate. However, the yield of the mono-potassium parapyruvate crystals was only 49.6% [2].

The alkaline treatment can induce pyruvic acid to form parapyruvate by an aldol addition with a pH value greater than 7.6 [14]. The higher the pH value of the solution, the more parapyruvate was produced [14]. After the alkaline treatment of pyruvic acid, a crude preparation solution of parapyruvate (CPSP) was prepared. We then recovered parapyruvate from the CPSP using acetone based on the principle of solvent crystallization [2,15]. However, the previous preparation conditions for the parapyruvate crystals synthesis were not within the optimal conditions because the yield should be further improved. Thus, the main purpose of this study was to explore the optimal conditions for the synthesis of pure parapyruvate with a high yield. We were also interested in revealing the major factors that affect the yield for the synthesized pure mono-potassium parapyruvate crystals.

The Taguchi method is an efficient approach for the multifactor optimizing conditions with low cost and low time consumption, and has been widely applied to different areas [16,17,18,19,20,21]. This method uses a special design of orthogonal arrays to dispense the control factors in a balanced manner for exploring the entire control factors through a small number of experimental trials, and converts the value obtained in the experimental results into a signal-to-noise ratio (S/N) to investigate the most optimal conditions [22]. For example, if a synthesis procedure contains four factors to influence the yield, and each factor is set to three different levels, 81 experiments are needed to evaluate all possible combinations of these factors, which is very time consuming. When the Taguchi L9 Orthogonal Array (L9 OA) is used instead of a full factorial experimental design, the determination of optimal conditions requires only 18 experiments.

Enzyme kinetic is usually used to monitor the relationship between substrate, inhibitor, and enzyme through the Michaelis and Menten equation [23]. The measurement steps involve adding different concentrations of the inhibitors and a fixed concentration of enzyme to enzyme reaction and then to measure the velocity of the enzyme reaction and further plot the experimental data to the Lineweaver–Burk plot to obtain the value of maximal velocity (Vmax) and the Michaelis constant (Km) [24]. Thus, the type of the inhibitor (competitive, non-competitive and uncompetitive) can be identified by the change of the value of Vmax and Km [24]. However, the inhibition of parapyruvate on the enzyme kinetic of the KGDHC hasn’t been studied to date. According to previous studies, parapyruvate is an analog of α-ketoglutarate (α-KG) [3], which should be a reversible competitive inhibitor of KGDHC. However, the study claimed that the inhibition of parapyruvate on KGDHC appears to be irreversible [3]. Therefore, another purpose of this study was to identify the parapyruvate-inhibited enzyme kinetic of KGDHC and to distinguish whether the inhibition is reversible or irreversible.

In this study, we applied Taguchi’s design method to determine the optimal conditions. We chose Taguchi L9 OA instead of a full factorial experimental design. For each preparation step, we selected the factors that may have a greater impact on the yield or purity of the synthetic parapyruvate as controllable factors. All of the controllable factors were set at three different levels. The optimal conditions were obtained by the controllable factors with the highest S/N ratio which was calculated by the yield or the recovery. In addition, we performed multiple solvent crystallization procedures until the purity of the parapyruvate crystals was greater than 99%. Furthermore, we used a commercial Enzyme-linked immunosorbent assay (ELISA) kit to investigate the enzyme kinetic of KGDHC inhibited by the co-treatment of parapyruvate.

## 2. Results and Discussion

### 2.1. Optimal Conditions of the CPSP Preparation

**Figure 1:**
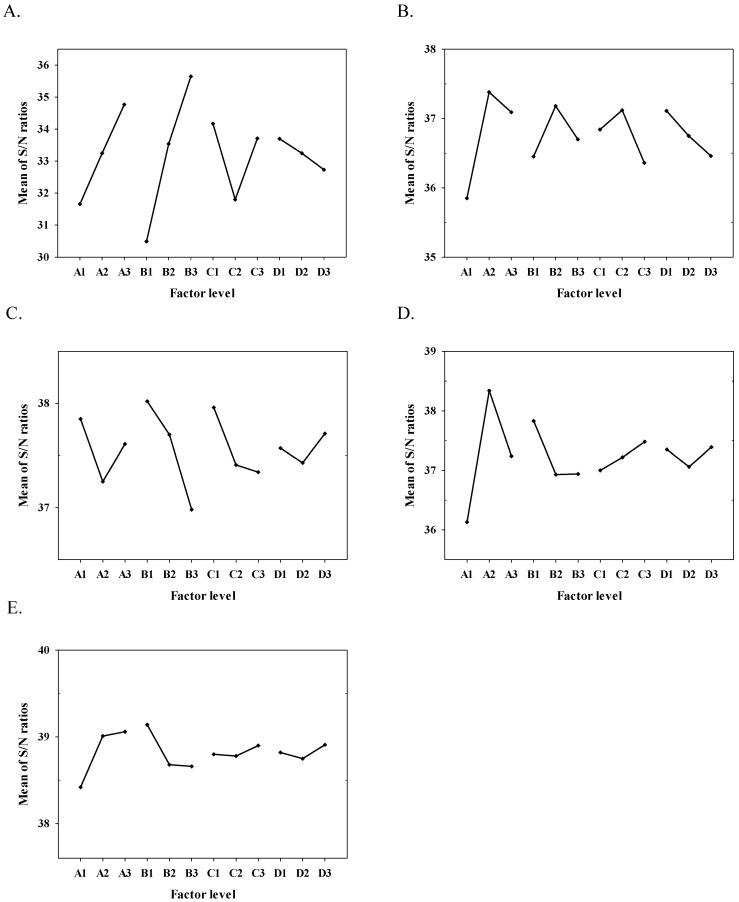
The main plot effects for the signal-to-noise (S/N) ratios of (**A**) the CPSP preparation, the first solvent crystallization by (**B**) ethanol, (**C**) methanol, (**D**) acetone and (**E**) the secondary solvent crystallization by ethanol.

For the processes of the CPSP preparation, the four controllable factors set with three levels are as shown in Table 1. After being analyzed by the HPLC and calculated by equation 1 in triplicate (n = 3), the results showed that the yield on average for different experimental conditions ranged from 32.9~68.0% (Table S1; at the top of the table (1) in the Supplementary Material). Where the yields were further converted into S/N ratio, values ranged from 30.34~36.65 (Table S1). We further used the S/N ratio to estimate the optimal conditions for the CPSP preparation. The average of the S/N ratios for each controllable factor are as shown in Table S2. Using the average of the S/N ratio, the delta value could be further obtained as the difference between the maximum and minimum of the S/N ratio, which could further estimate the effective order of the controllable factors [18]. The results showed that factor B (i.e., the pH of dimerization reaction) had the most substantial delta value of 5.16, suggesting that factor B was the most effective factor on the yield at this stage for the preparation of CPSP. On the other hand, factor D with the slightest delta value of 0.97 was the less effective factor. The results showed that the effective order of controllable factors for the CPSP preparation were ranked from (B) the pH of dimerization reaction > (A) the concentration of pyruvic acid solution > (C) the temperature of the dimerization reaction > (D) the pH of stop reaction. Based on the highest S/N ratio among each level for each factor, the optimal conditions were A3, B3, C1 and D1 (Figure 1A and Table S2). The yield of the optimized conditions of A3, B3, C1 and D1 at this stage were further confirmed, and the data as shown in Table S1 and the yield could be extended to 85.1%.

### 2.2. Optimal Conditions for First Solvent Crystallization to Recover the Parapyruvate Crystals from the CPSP

In this study, we selected ethanol, methanol, and acetone as the first solvent for crystallization, respectively. The recovery of the parapyruvate crystals for the above three solvents were calculated by Equation (3), i.e., R1 (%). The results showed that the recovery of the first crystallization by ethanol, methanol, and acetone varied between 56.6~76.7%, 69.7~86.0% and 60.6~89.0%, respectively (Table S1). In addition, the means of S/N ration of four controllable factors in ethanol, methanol, and acetone are as shown in Table S2 and Figure 1B–D. According to the highest values of the S/N ratio among each level for each factor, the optimal conditions for each solvent could be obtained and the recovery of the optimal conditions of ethanol, methanol, and acetone were 77.2, 86.8, and 89.3%, respectively (Table S1). All of these results suggested that the optimal conditions of the solvent crystallization were A2, B1, C3, and D3 using acetone (Figure 1D). In addition, the delta values varied according to the solvents used, and the highest delta value of the first solvent crystallization was acetone (delta value = 2.21; Table S2), which means that the type of used solvent significantly affected the yield, and the acetone was the best choice for the first solvent crystallization. When acetone was used for the first solvent crystallization, the factor that had the greatest impact on parapyruvate recovery was the volume ratio of CPSP and acetone. In addition, the purity of the produced parapyruvate crystals from the first crystallization by acetone ranged from 69% to 90.8, which didn’t reach our goal with a purity of >99% (Table S1). Thus, the whole processes needed a second solvent crystallization to recover a pure parapyruvate.

### 2.3. Optimal Conditions for the Secondary Solvent Crystallization by Ethanol

The secondary crystallization was conducted to account for improving the purity of the impure parapyruvate crystals produced by the first solvent crystallization (the impure parapyruvate after the first crystallization is now denoted as IPFC). Due to the saturated concentration of the parapyruvate aqueous solution that was approximately 150 mM, the concentration was used to test the optimal conditions for the second solvent crystallization. Furthermore, because we had preliminary tested and found that ethanol was the best solvent for the secondary solvent crystallization, we used ethanol as the solvent for the secondary solvent crystallization procedures. The results showed that the obtained purity in nine experimental trials were all higher than 99%. On the other hand, recovery ranged between 80.8~93.0% and was further transformed to the S/N ratio (Table S1). The influential order of controllable factors for the secondary solvent crystallization judged by the delta value are as shown in Table S2, which were ranked from (A) the volume ratio of 150 mM IPFC solution and ethanol > (B) the standing temperature > (D) the mixing time > and (C) the standing time. Consequently, the optimum for secondary solvent crystallization by ethanol would be the highest S/N ratio which were A3, B1, C3 and D3 (Figure 1E) with a purity of 99.8% and a recovery of 93.2% (Table S1).

### 2.4. The Final Yield and Purity for Synthesizing Parapyruvate by the Optimum Conditions

The obtained final optimal conditions were (1) A3B3C1D1 for the CPSP preparation, (2) A2B1C3D3 for the first solvent crystallization by acetone, and (3) A3B1C3D3 for the secondary solvent crystallization by ethanol (Table 2). The obtained whole optimal procedures were summarized as follows: 2 M pyruvic acid solution adjusted its pH to 12 and stood in 4 °C for 15 min to allow for the dimerization of pyruvic acid and then terminated the dimerization by adjusting pH to 2 so as to obtain the CPSP. The CPSP was further added with a 2.5 times volume of acetone and vortexed for 60 s, then stood at −20 °C for 24 h, thereby obtaining the parapyruvate crystals. Furthermore, 150 mM IPFC solution was added 12 times the volume of ethanol and vortexed for 60 s, then stood at −20 °C for 24 h, for conducting the second solvent crystallization to improve the purity of the parapyruvate crystals. Using the whole optimal conditions, we confirmed the present modified method by HPLC and the results showed that a yield of 72.8% and a purity of 99.8% was obtained (Table 2). To the best of our knowledge, this is the first study in the literature to reveal all of the optimal conditions for the pure parapyruvate synthesis and revealed the main factors that affect the synthesis of parapyruvate. Compared to our previous method [2], the overall yield of the present method was greatly improved from approximately 50% to 72.8%, which improved the yield by more than 20%. Since the yield of the CPSP under optimal conditions is only 85.1%, the total yield of the present method can reach 72.8%, which is already very high. In addition, the present method uses a second solvent crystallization by ethanol, which showed that the pure parapyruvate crystals could be obtained. Thus, the previous washing procedure by methanol can now be omitted in the present method. The obtained method can be applied to other preparation methods based on the principle of aldol addition so as to synthesize the chemicals in an alkali environment, as well as any solvent crystallization preparation process to prepare the crystals.

### 2.5. The Inhibition of Parapyruvate on KGDHC Enzyme Kinetic

**Figure 2:**
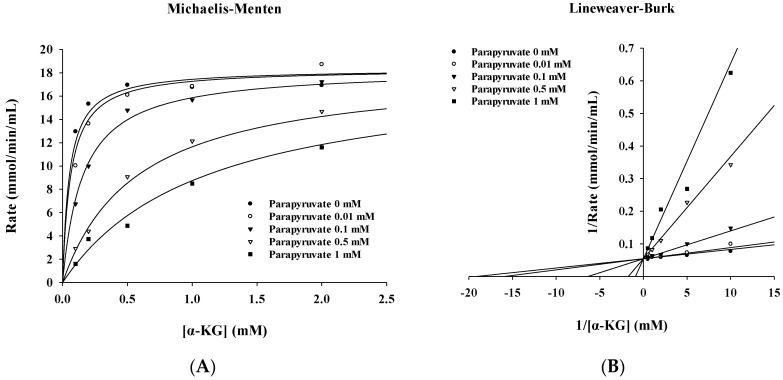
The inhibition kinetics of parapyruvate on the KGDHC. (**A**) Michaelis Menten plot. (**B**) Lineweaver–Burk plot.

**Figure 3:**
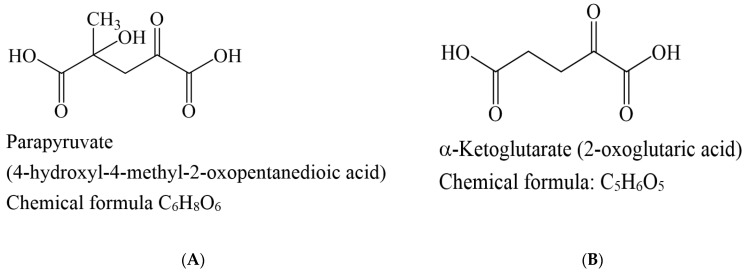
The structures of (**A**) parapyruvate and (**B**) α-ketoglutarate.

The enzyme kinetics of KGDHC in the presence of the inhibitor (parapyruvate 0~1 mM) were determined from Michaelis-Menten (Figure 2A) and Lineweaver-Burk plots (Figure 2B). The concentration-dependent rates measured in Figure 2 are as shown in Table S3 in the Supplementary Material. Values of Vmax and Km were calculated by fitting the slope of linear regression in the Michaelis-Menten formula and are listed in Table 3. As (1) the inhibitor concentration is increased and the Km value is also increased without affecting the value Vmax, (2) the Lineweaver–Burk plot shows the intersection of straight lines with different slopes on the *Y* axis, which were characteristic of the reversible competitive inhibitor [25]. Consequently, the kinetic analyzed data demonstrated that the inhibition of the KGDHC by parapyruvate was typically a concentration-dependent reversible competitive type rather than an irreversible inhibitor. As shown in Figure 3A,B, the chemical structure of the parapyruvate and α-ketoglutarate are quite similar.

## 3. Materials and Methods

### 3.1. Materials

All chemicals used were analytical grade. KOH and HCl, was purchased from Merck (Darmstadt, Germany). Pyruvic acid was purchased from Alfa Aesar (Heysham, England). Sodium pyruvate, β-nicotinamide adenine dinucleotide hydrate, NAD^+^, thiamine pyrophosphate (TPP) and coenzyme A sodium salt hydrate (CoA) were obtained from Sigma Chemical Corp. (St. Louis, MO, USA). Methanol was purchased from Spectrum Chemical Manufacturing Corp. (New Brunswick, NJ, USA). Acetone was purchased from Macron Fine Chemicals™ (Center Valley, PA, USA). Ethanol was purchased from J.T.Baker^®^ Chemicals (Phillipsburg, NJ, USA).

### 3.2. Determination of the Optimal Conditions for Synthesizing Parapyruvate

The method for producing mono-potassium parapyruvate crystals was modified from our previous study [2]. In our previous method, the procedures contained three main steps including the preparation of CPSP, the solvent crystallization, and the produced crystal washing with methanol. In this study, the synthesized method was modified as being different in the three main steps: (1) the preparation of CPSP, (2) the first solvent crystallization and (3) the secondary solvent crystallization. The methods for determining the optimal conditions of the modified method in this study for each step are described in detail as follows.

#### 3.2.1. Determine the Optimal Conditions for the Preparation of CPSP

Pyruvic acid solution in deionized water was prepared, and the alkalization with a 10 M KOH solution to promote the dimerization of pyruvic acid by adjusting the pH over 8 was undertaken. After 15 min of standing time for the dimerization, we performed an acidification with 10 M HCl solution to terminate the dimerization by adjusting the pH to lower than 4, and then obtained the CPSP. We chose four controllable factors, and each controllable factor was set at three levels (Table 1) which, respectively, were (A) the concentration of raw material of pyruvic acid solution: 0.5, 1, and 2 M, (B) the pH of polymerization reaction: 8, 10, and 12, (C) the temperature of polymerization reaction: 4, 25, and 37 °C, and (D) the pH of stop reaction: 2, 3 and 4 by applying Taguchi L9 OA to design the experimental trials.

#### 3.2.2. Determine the Optimal Conditions for the First Solvent Crystallization to Recover Parapyruvate from the CPSP

The parapyruvate in the CPSP was recovered via a solvent crystallization method. The CPSP was added more than two times to the CPSP volume of solvent and mixed together for more than 10 s, then stood for more than six hours at a temperature below 25 °C to recover the parapyruvate form crystals. The crystals were dried in an oven at 50 °C. For comparing the ability of recovery, three types of solvent were selected for the first solvent crystallization, which included ethanol, methanol, and acetone. Each solvent was a conducted solvent crystallization, respectively, with four controllable factors which was set at three levels including (A) the volume ratio of CPSP and solvent: 1:2, 1:2.5 and 1:3, (B) the standing temperature for the first solvent crystallization: −20, 4 and 25 °C, (C) the standing time for the first solvent crystallization: 6, 12 and 24 h, and (D) the mixing time for the first solvent crystallization: 10, 30 and 60 s (Table 1).

#### 3.2.3. Determine the Optimal Conditions for the Secondary Solvent Crystallization to Recover the Pure Parapyruvate from the Impure Crystals

Because the purity of the parapyruvate crystals was not high enough after crystallization in the first solvent, we thus used ethanol for a second solvent crystallization to obtain the high-purity parapyruvate crystals. In detail, the obtained IPFC was resolved in pure water so as to obtain 150 mM of IPFC solution. The IPFC solution (150 mM) was added to more than nine times the volume of ethanol and mixed together for more than 10 s, then stood for more than six hours at a temperature lower than 25 °C to produce the parapyruvate crystals with high purity. For this procedure, four controllable factors were selected and each controllable factor was set at three levels, which included (A) the volume ratio of IPFC to ethanol: 1:2, 1:2.5 and 1:3, (B) the standing temperature for the second solvent crystallization: −20, 4 and 25 °C, (C) the standing time for the second solvent crystallization: 6, 12 and 24 h, and (D) the mixing time for the second solvent crystallization: 10, 30 and 60 s (Table 1).

### 3.3. HPLC Analysis

Parapyruvate was determined using a reverse-phase high-performance liquid chromatography (HPLC) system consisting of the HP Series 1050 Pumping Systems (Palo Alto, CA, USA), the HP 1050 Series Online Degasser, Athena C18 column (4.6 mm × 250 mm, 5 μm), and the HP 1050 Series Variable Wavelength Detector. The analysis method was referred to our previous study [2]. The mobile phase was 0.02% sulfuric acid solution, and the analysis was achieved by using an isocratic elution for 10–30 min at a flow rate of 1 mL/min. Absorbent signals were detected with a UV wavelength of 220 nm. The sample loop was 20 μL. The column temperature was set at 25 °C.

### 3.4. Determination of the Yield, Recovery, and Purity of the Synthesized Parapyruvate

The yield (%) for the CPSP preparation was calculated by the following equation:

Yield (%) = W_1_ ÷ W_0_ × 100% = C_0_ × V_0_ ÷ W_0_ × 100% (1)

where W_0_ was the weight of the pyruvic acid (mg), the reactants required for the polymerizing reaction; W_1_ was the weight of the parapyruvate (mg) contained in the CPSP; C_0_ was the concentration of parapyruvate (mg/L) in the CPSP determined by the HPLC method; V_0_ was the volume of the CPSP (L).

The purity equation for the first solvent crystallization (P1) was expressed as:

P_1_ (%) = {[C_1_ (mg/L) × 1 L × (214.2 ÷ 176.1)] ÷ [W_2_ (mg)]} × 100% (2)

where W_2_ was the parapyruvate crystals (mg) obtained from the first solvent crystallization; C_1_ was the concentration of parapyruvate crystals (mg/L) dissolved in the 1 L solution determined by the HPLC; 214.12 was the molecular weight of mono-potassium parapyruvate; 176.12 was the molecular weight of parapyruvate.

For the solvent crystallization, a higher recovery means the developed method would have a higher yield. Thus, we here used the recovery to estimate the optimal conditions for the solvent crystallization processes. The recovery equation for the first solvent crystallization (R_1_) was calculated as below:

R_1_ (%) = W_2_ (mg) × P_1_ (%) × (176.1 ÷ 214.2) ÷ W_1_ (mg) × 100% (3)

The purity equation for the second solvent crystallization (P_2_) was determined by the following equation:

P_2_ (%) = {[C_2_ (mg/L) × 1 L × (214.2 ÷ 176.1)] ÷ [W_3_ (mg)]} × 100% (4)

where C_2_ was the concentration of the parapyruvate crystals acquired from the secondary solvent crystallization that were dissolved in 1 L of pure water determined by the HPLC; W_3_ was the parapyruvate crystals (mg) obtained from secondary solvent crystallization.

The recovery equation for the second solvent crystallization (R_2_) was calculated as below:

R_2_ (%) = [W_3_ (mg) × P_2_ (%)] ÷ [W_2_ (mg) × P_1_ (%)] × 100% (5)

### 3.5. Taguchi Design of Experiment

Since each condition was analyzed in triplicate (n = 3), depending on the Taguchi approach, the experimental results should be transformed to the S/N ration to assess the optimal condition. In general, there are three types of S/N ratio analysis available: (1) lower is better (LB), (2) nominal is best (NB), and (3) higher is better (HB) [26]. Since the target of this study is to obtain the parapyruvate crystals with the greatest yield and a purity higher than 99%, yield or recovery were selected to convert to the S/N ratio to further evaluate the optimum. Moreover, the optimal level of the controllable factors is the level with the highest S/N ratio, which is given as the below equation:

(6) $$S/N=-10log_{10}\left[\frac{1}{n}\overset{n}{\underset{i=1}{\sum }}\left(\frac{1}{y_{i}{}^{2}}\right)\right]$$

where *n* is the number of repetitions under the same experimental conditions, and *y_i_* represents the measurement results i.e., the yield or recovery.

### 3.6. KGDHC Enzyme Kinetic Assay

The enzyme activity assay was performed by using a commercial α-Ketoglutarate Dehydrogenase Activity Colorimetric Assay Kit (Biovision, K678-100, Milpitas, CA, USA). In the 100μL reaction mixture which contains one of the indicated concentrations of α-KG and parapyruvate, and 15μL KGDHC mixture which included NAD^+^ 0.5 mM, TPP 0.2 mM and CoA 0.04 mM, 2 μL KGDHC developer and 3 μL KGDHC positive control, and adjusted the final volume to 100 µL with the KGDH assay buffer. After incubation for 30 min, the absorbance at 450 nm was detected. The substrate α-KG was set at concentrations of 0, 0.1, 0.2, 0.5, 1 and 2 mM and the inhibitor of parapyruvate was set at concentrations of 0, 0.01, 0.1, 0.5, 1, 5, 10 and 20 mM. The kinetic parameters, Michaelis Menten plot, and Lineweaver–Burk plot were analyzed by using the sigma plot 14.0 software (Systat Software, Inc., San Jose, CA, USA).

## 4. Conclusions

In this study, the optimal conditions were determined for synthesizing the parapyruvate crystals with a high yield and high purity by using the Taguchi method L9 OA. The main processes of the developed method included (1) the preparation of CPSP, (2) the crystallization in the first solvent, and (3) the crystallization in the second solvent. The results showed that a higher pH and pyruvic acid solution concentration in the reaction of alkaline dimerization had the greatest effect on the yield of parapyruvate in the CPSP. For the first solvent crystallization procedures, the type of used solvent much affected the yield and the acetone was the best choice for this step. For the first and second solvent crystallization procedures, the volume ratio between the used solvent and CPSP or IPFC had the most impact on the yield of the pure parapyruvate crystals. Finally, the whole optimal processes can yield 72.8% of the parapyruvate crystals with a purity of 99.8%. Additionally, the study distinguishes that the parapyruvate is a reversibly competitive inhibitor for the KGDHC.

## 5. Patents

This method has obtained the patent of Taiwan Invention No. I655184 “The production method of 2-hydroxy-2-methyl-4-oxopentanedioic acid” (the patent owner: Nae-Cherng Yang and Inn Lee; the Application granted date: 1/4/2019).

## Figures and Tables

**Table 1:** The controllable factors and levels for the CPSP preparation, first solvent crystallization and secondary solvent crystallization.

(1) CPSP * Preparation				
Symbol	Factor	Level		
		1	2	3
A	Concentration of pyruvic acid solution (M)	0.5	1	2
B	pH of dimerization reaction	8	10	12
C	Temperature of dimerization reaction (°C)	4	25	37
D	pH of stop reaction	2	3	4
**(2) First Solvent Crystallization**				
Symbol	Factor	Level		
		1	2	3
A	Volume ratio of CPSP: solvent	1:2	1:2.5	1:3
B	Standing temperature (°C)	−20	4	25
C	Standing time (hour)	6	12	24
D	Mixing time (seconds)	10	30	60
**(3) Secondary Solvent Crystallization**				
Symbol	Factor	Level		
		1	2	3
A	Volume ratio of IPFC solution **: ethanol	1:9	1:10	1:12
B	Standing temperature (°C)	−20	4	25
C	Standing time (hour)	6	12	24
D	Mixing time (seconds)	10	30	60

* CPSP: The crude preparation solution of the parapyruvate through conducting the alkalization to pyruvic acid aqueous solution; ** The concentration of IPFC solution is 150 mM. The IPFC means the impure parapyruvate after the first crystallization.

**Table 2:** Confirmation of the yield and purity of the modified method for producing the pure parapyruvate crystals with the optimal conditions.

Run	CPSP Preparation	First Solvent Crystallization by Acetone	Secondary Solvent Crystallization by Ethanol	Yield (%)	Purity (%)
Optimum	A3B3C1D1	A2B1C3D3	A3B1C3D3	72.8	99.8

Factors and levels are obtained from Table S1.

**Table 3:** Kinetic parameters of the KGDHC inhibition by the parapyruvate.

Inhibitor (mM) *	V_max_ (nmol/min/mL)	K_m_ (mM)	Inhibitor Type
0	18.5154	0.0324	Competitive **
0.01	18.9913	0.0862	
0.1	18.7824	0.1698	
0.5	19.1352	0.5875	
1	18.6393	1.0747	

* Inhibitor is the pure parapyruvate crystals; ** Competitive inhibitor (V_max_ unchanged while the K_m_ increased).

## Data Availability

The data presented in this study are available on request from the corresponding author.

## Supplementary Materials

The following supporting information can be downloaded at: https://www.mdpi.com/article/10.3390/molecules27061870/s1; Table S1: L9 OA design of the experiments for the CPSP preparation, and the first and secondary solvent crystallization by different solvents; Table S2: Response table of the CPSP preparation, and first and secondary solvent crystallization by the different solvents; Table S3. The kinetic data of the KGDHC enzyme inhibited by the parapyruvate.

## Sample Availability

Samples of the parapyruvate are available from the authors, if there is a partnership with us.
