# Supplementary material for: Using Taguchi Method to Determine the Optimum Conditions for Synthesizing Parapyruvate
Source: Molecules. 2022 Mar 14;27(6):1870. doi: 10.3390/molecules27061870 (PMC8954423; doi:10.3390/molecules27061870)
Supplement: Supplementary file 1 [file molecules-27-01870-s001.zip › molecules-1623434-supplementary.pdf]

Supplementary Materials

Table S1. L9 OA design of the experiments for the CPSP preparation, and the first and secondary solvent crystallization by different solvents.

Run	Factors				Yield (%)	S/N ratio	
**(1) CPSP preparation**							
	A	B	C	D			
1	1	1	1	1	32.9	30.34	
2	1	2	2	2	33.8	30.57	
3	1	3	3	3	50.5	34.06	
4	2	1	2	3	26.9	28.58	
5	2	2	3	1	53.2	34.52	
6	2	3	1	2	68.0	36.65	
7	3	1	3	2	42.4	32.54	
8	3	2	1	3	59.8	35.53	
9	3	3	2	1	64.9	36.24	
Optimum	3	3	1	1	85.1	38.60	
**(2) First solvent crystallization by ethanol**							
Run	Factors				Purity (%)	Recovery (%)	S/N ratio
	A	B	C	D			
1	1	1	1	1	67.1	62.6	35.93
2	1	2	2	2	74.3	67.4	36.57
3	1	3	3	3	87.8	56.6	35.05
4	2	1	2	3	71.2	71.5	37.08
5	2	2	3	1	76.1	76.8	37.71
6	2	3	1	2	84.9	73.7	37.35
7	3	1	3	2	65.4	65.5	36.32
8	3	2	1	3	72.2	72.9	37.25
9	3	3	2	1	79.3	76.7	37.69
Optimum	2	2	2	1	75.2	77.2	37.75
**(3) First solvent crystallization by methanol**							
Run	Factors				Purity (%)	Recovery (%)	S/N ratio
	A	B	C	D			
1	1	1	1	1	88.8	86.0	38.69
2	1	2	2	2	90.7	76.7	37.69
3	1	3	3	3	98.2	72.2	37.17
4	2	1	2	3	78.1	76.6	37.68
5	2	2	3	1	80.8	72.0	37.14
6	2	3	1	2	83.3	70.1	36.91
7	3	1	3	2	74.7	76.7	37.69
8	3	2	1	3	87.2	82.0	38.27
9	3	3	2	1	94	69.7	36.86
Optimum	1	1	1	3	94.7	86.8	38.77
**(4) First solvent crystallization by acetone**							
Run	Factors				Purity (%)	Recovery (%)	S/N ratio
	A	B	C	D			
1	1	1	1	1	81.7	67.7	36.61
2	1	2	2	2	74.1	60.6	35.65
3	1	3	3	3	90.8	64.1	36.14
4	2	1	2	3	76.9	89.0	38.99
5	2	2	3	1	74.1	83.1	38.39
6	2	3	1	2	82.4	76.2	37.64
7	3	1	3	2	69	78.6	37.91

8	3	2	1	3	72.5	68.9	36.76
9	3	3	2	1	78.3	71.1	37.04
Optimum	2	1	3	3	81.1	89.3	39.02

(5) Secondary solvent crystallization by ethanol

Run	Factors				Purity (%)	Recovery (%)	S/N ratio
	A	B	C	D			
1	1	1	1	1	99.7	86.1	38.70
2	1	2	2	2	99.7	80.8	38.15
3	1	3	3	3	99.8	83.3	38.41
4	2	1	2	3	99.8	92.8	39.35
5	2	2	3	1	99.9	88.4	38.93
6	2	3	1	2	99.9	86.5	38.74
7	3	1	3	2	99.9	93.0	39.37
8	3	2	1	3	99.7	88.7	38.96
9	3	3	2	1	99.9	87.5	38.84
Optimum	3	1	3	3	99.8	93.2	39.39

* CPSP: The crude preparation solution of the parapyruvate through conducting alkalization to the pyruvic acid aqueous solution.

Table S2. Response table of the CPSP preparation, and first and secondary solvent crystallization by the different solvents.

Level	Factors			
**(1) CPSP * preparation**				
	A	B	C	D
1	31.66 **	30.49	34.17	33.70
2	33.25	33.54	31.80	33.25
3	34.77	35.65	33.71	32.73
Delta	3.12	5.16	2.37	0.97
Rank	2	1	3	4
**(2) First solvent crystallization by ethanol.**				
Level	A	B	C	D
1	35.85	36.45	36.84	37.11
2	37.38	37.18	37.12	36.75
3	37.09	36.70	36.36	36.46
Delta	1.53	0.73	0.76	0.65
Rank	1	3	2	4
**(3) First solvent crystallization by methanol.**				
Level	A	B	C	D
1	37.85	38.02	37.96	37.57
2	37.25	37.70	37.41	37.43
3	37.61	36.98	37.34	37.71
Delta	0.60	1.04	0.62	0.28
Rank	3	1	2	4
**(4) First solvent crystallization by acetone.**				
Level	A	B	C	D
1	36.13	37.83	37.00	37.35
2	38.34	36.93	37.22	37.06
3	37.24	36.94	37.48	37.39
Delta	2.21	0.90	0.47	0.33
Rank	1	2	3	4
**(5) Secondary solvent crystallization by ethanol.**				
Level	A	B	C	D
1	38.42	39.14	38.80	38.82
2	39.01	38.68	38.78	38.75
3	39.06	38.66	38.90	38.91
Delta	0.64	0.48	0.12	0.16
Rank	1	2	4	3

* CPSP: The crude preparation solution of the parapyruvate through conducting alkalization to the pyruvic acid aqueous solution.
** Values are an average of the S/N ratios which are obtained from Table S1 by the Equation 6.

Table S3. The kinetic data of the KGDHC enzyme inhibited by the parapyruvate.

α-KG * (mM)	Parapyruvate (mM)				
	**0**	**0.01**	**0.1**	**0.5**	**1**
0.1	12.97 **	10.04	6.74	2.92	1.60
0.2	15.34	13.64	9.99	4.40	3.72
0.5	16.95	16.10	14.79	9.08	4.87
1	16.77	16.84	15.67	12.14	8.49
2	16.93	18.73	17.23	14.68	11.60

*α-KG: α-ketoglutarate.

** Values are the reaction rate.
